# Latent patterns of rumination and hopelessness on self-harm behaviors in adolescence

**DOI:** 10.1186/s13034-026-01029-0

**Published:** 2026-01-29

**Authors:** Mengting Wang, Shuwen Chen, Jingyi Gao, Cheng Cheng, Li Yang, Hui Ai

**Affiliations:** 1 School of Education, Tianjin University, Tianjin, China; 2 Institute of Applied Psychology, Tianjin University, Tianjin, China; 3 Academy of Medical Engineering and Translational Medicine, Tianjin University, Tianjin, China; 4 Laboratory of Suicidal Behavior Research, Tianjin University, Tianjin, China; 5 School of Psychology, Shenzhen Key Lab of Affective and Social Cognitive, Shenzhen University, Shenzhen, China

**Keywords:** Rumination, Hopelessness, Self-harm behaviors, Latent profile analysis

## Abstract

**Objectives:**

Self-harm behaviors among adolescents, including suicide attempts (SA) and non-suicidal self-injury (NSSI), present critical public health challenges globally. While past-oriented rumination and future-oriented hopelessness are established core dimensions of maladaptive self-cognition, their distinct associations with different forms of self-harm remain poorly characterized.

**Methods:**

Using latent profile analysis, we investigated the heterogeneous profiles of rumination and hopelessness in a sample of 951 adolescents (M_age_: 16.58; male: 420). We further examined how these latent profiles differ in their associations with SA and NSSI, controlling for general affective symptoms.

**Results:**

Three distinct cognitive profiles were identified: high rumination–high hopelessness (*n* = 77, 8.09%), moderate rumination–moderate hopelessness (*n* = 531, 55.84%), and low rumination–low hopelessness (*n* = 343, 36.07%). Specifically, the high rumination–high hopelessness profile emerged as being uniquely associated with SA, even after controlling for affective symptoms. NSSI was related to the severity of depressive symptoms, but not related to the specific cognitive profiles.

**Conclusion:**

Our study provides novel insights that the interaction between rumination and hopelessness generates distinct cognitive phenotypes, which show different associations with SA and NSSI. These findings address a key theoretical gap in self-harm mechanisms and suggest the need to reshape prevention paradigms by enabling phenotype-specific interventions targeting cognitive constrictions for at-risk youth.

**Supplementary Information:**

The online version contains supplementary material available at 10.1186/s13034-026-01029-0.

## Introduction

Self-harm behaviors comprise a spectrum of injurious behaviors directed at oneself, ranging from non-suicidal self-injury (NSSI), which is not intended to result in death, to suicide attempts (SA), which involve clear suicidal intent, and ultimately to those who died by suicide [1]. Globally, more than 720,000 individuals die by suicide each year [2], making it the second leading cause of death among adolescents and young adults [3]. Recent epidemiological research indicates that NSSI is highly prevalent, with an estimate lifetime prevalence of approximately 22.0% among adolescents worldwide, and a recurrence rate of 20.3% [4]. The burden is particularly pronounced in China, where the lifetime prevalence is even higher, estimated at 24.7% among youths [5]. With detection rates of 22.8% for NSSI and 2.9% for SA among Chinese high school students [6], these behaviors constitute major public health concerns among Chinese adolescents. Clarifying the key factors associated with SA and NSSI in adolescents may inform the development of early intervention and targeted prevention strategies.

Adolescence is a critical period for the formation of self-identity. During this developmental stage, individuals integrate past, present, and future self-related experiences to construct a coherent sense of self, which significantly influences psychological well-being [7]. Self-harm behaviors are understood as a dynamic process influenced by temporal factors [8]. A recent study has identified that, among various predictors of suicide behaviors, hopelessness regarding the future constituted the strongest risk factor [9]. Furthermore, rumination on past events has also been identified as an important transdiagnostic predictor of mental disorders [10]. Therefore, incorporating both past-oriented and future-oriented psychological constructs may enhance our understanding of the cognitive profiles associated with adolescent self-harm behaviors. Moreover, while the development of SA and NSSI in adolescents may co-occur and share certain affective and behavioral features, they are theoretically differentiated by the presence of suicidal intent, the temporal orientation of the underlying motivation (e.g., future-oriented hopelessness in SA vs. immediate affect regulation in NSSI), and the development of acquired capability for suicide [11–13]. Distinguishing these two different types of self-harm behaviors based on their respective self-cognitive constructs is crucial for informing precise and individualized interventions.

Hopelessness is a cognitive construct closely associated with future-oriented thinking. The core components of hopelessness include the abandonment of goals and aspirations, as well as negative expectations about the future [14]. Hopelessness has been found to be associated with both suicide behaviors and NSSI, as individuals with higher levels of hopelessness exhibited an increased propensity for NSSI [15, 16]. Rumination is a maladaptive form of cognitive processing and an ineffective emotion regulation strategy [17]. Characterized by repetitive focus on past events and the causes and consequences of negative emotions, meta-analytic evidence suggests that rumination predicts both short-term and life-time SA among individuals with depressive disorders as well as in non-clinical populations [18]. Furthermore, studies have found rumination to be positively associated with the frequency of NSSI [19]. Individuals exhibiting higher levels of rumination tend to engage in more frequent NSSI when experiencing depressive symptoms [20]. A previous study has found that rumination moderated the mediating role of hopelessness in the relationship between self-criticism and NSSI [21]. Nonetheless, no study has jointly examined the roles of past-oriented rumination and future-oriented hopelessness in both SA and NSSI. Hopelessness and rumination have also been identified to have transdiagnostic predictive effects on anxiety and depression [22, 23]. However, it remains unclear whether the association between combined hopelessness-rumination and self-harm behaviors were independent of depressive and anxiety symptoms.

In summary, the formation of self-identity in adolescents is associated with cognitive processing directed toward both the past and the future. Our study aims to identify the core features of adolescents’ future-oriented hopelessness and past-oriented rumination, and to examine how these features are associated with different self-harm behaviors. We hypothesized that adolescents will exhibit distinct latent profiles characterized by different combinations of rumination and hopelessness levels, and these profiles will show differential associations with SA and NSSI, reflecting potentially distinct risk pathways. To this end, we will use latent profile analysis to identify the potential categories of the combined traits of rumination and hopelessness in adolescents. Then, based on these categories, we will use hierarchical logistic regression to analyze the relationship of these combined traits with different self-harm behaviors. To isolate the specific contribution of latent profiles of rumination and hopelessness beyond affective symptoms, all key associations will be statistically adjusted for depressive and anxiety symptom severity.

## Method

### Participants

Participants were selected from first-grade and second-grade students at a high school in western China. After screening for response quality (inconsistency between positive/negative questions and responses, *N* = 40), 951 valid questionnaires were obtained, accounting for 95.96% of the total. Of those, 420 (44.16%) were male and 531 (55.84%) were female.

This study received ethical approval from the University Ethical Review Board. Informed consent was obtained from the participants and their primary caregivers prior to conducting the study. Consistent with institutional and school policies, any participants who endorsed high-risk items during the screening process were referred to the school for appropriate follow-up and psychological support.

### Measures

#### Ruminative responses scale (RRS)

Rumination was assessed using the revised Chinese version of the Ruminative Responses Scale (RRS), a 22-item self-report measure assessed on a 4-point Likert scale. This scale comprises three subscales: brooding, depressive symptoms, and reflection. This adaptation has been previously administered in samples of Chinese high school and undergraduate students, demonstrating established reliability and validity within this socio-cultural context [24, 25]. To minimize the issue of multicollinearity arising from the significant variance shared by the overall RRS and measures of affective symptoms, only the Brooding and Reflection subscales were selected as indicators for the LPA. This selection aligns with the empirically supported distinction that these two subscales represent the core, maladaptive and adaptive cognitive components of rumination, respectively, as validated by Treynor et al. [26]. In our sample, the Cronbach’s alpha coefficient for the scale was 0.96.

#### Beck Hopeless Scale (BHS)

Hopelessness, defined as an individual’s negative expectations regarding the future, was measured using the Chinese version of the Beck Hopelessness Scale (BHS) [27]. This adaptation has demonstrated strong psychometric properties in large samples of Chinese adolescents, with a previously reported internal consistency of α = 0.85, supporting its suitability for use with Chinese youth. The scale has 20 entries and measures three dimensions: feelings about the future, loss of motivation, and future expectations. Total scores range from 0 to 20, with higher scores indicating higher levels of hopelessness. Since this study examined cognitive processing in the temporal dimension, the feelings about the future and future expectations were used as observational variables in the latent profile analysis because they were closely related to the future. In this study’s sample, Cronbach’s alpha coefficient was 0.72.

#### State-Trait Anxiety Inventory (STAI)

Anxiety symptoms were assessed using the Chinese version of State-Trait Anxiety Inventory (STAI), which has been validated for secondary school students in China [28]. The first 20 items assess state anxiety (S-AI), reflecting individuals’ transient experiences of stress and worry at a given moment, while the remaining 20 items assess trait anxiety (T-AI), representing a relatively stable predisposition to experience anxiety. Each item is rated on a 4-point scale, ranging from 1 (“not at all”) to 4 (“very much so”), yielding total scores between 20 and 80. Higher scores indicate greater levels of state anxiety. In our study, only the state anxiety subscale was used to assess adolescents’ current anxiety levels. The Cronbach’s alpha coefficient for this subscale was 0.87.

#### Beck Depression Inventory-II (BDI-II)

Depressive symptoms over the past week were assessed using the Chinese version of the Beck Depression Inventory-II (BDI-II). This scale has demonstrated high reliability when administered in samples of Chinese middle-school students [29]. The questionnaire comprises 21 items, each rated on a 4-point scale ranging from 0 to 3. The total score, calculated by summing all item responses, ranges from 0 to 63. Based on established cut-off scores, a total score of 0–13 indicates minimal or no depression, 14–19 indicates mild depression, 20–28 indicates moderate depression, and 29–63 indicates severe depression. In the current sample, the Cronbach’s alpha coefficient for the scale was 0.91.

#### Self-Harm Behaviors Assessment

Non-suicidal self-injury (NSSI) and suicide attempts (SA) were assessed through explicit questions. NSSI was evaluated by asking participants: “Have you ever intentionally hurt yourself without the intent to die?” Similarly, SA was assessed with the question: “Have you ever attempted suicide or engaged in any behavior that could be considered suicidal?”. Both SA and NSSI were measured as life-time behaviors. Single-item assessment has been shown to be a reasonable method for capturing lifetime SA/NSSI [30].

### Data analysis

Demographics data were analyzed by using SPSS version 26.0. Latent Profile Analysis (LPA), robust three-step approach (R3STEP) and Bolck, Croon, and Hagenaars’s method (BCH) analyses were conducted in Mplus version 8.3. Subsequent hierarchical logistic regression based on LPA derived categories were performed using R version 4.4.1.

LPA was used to explore joint patterns of adolescent rumination (i.e., brooding and reflection subscales of the RRS) and hopelessness about the future (i.e., feelings about the future and future expectations from BHS). Prior to conducting LPA, all variables were standardized using z-score transformation. We used the MLR estimator, with 200 random starts and 50 final optimizations to minimize the risk of local maxima. The model was estimated using the default Mplus random seed and default convergence criteria, and all solutions converged normally without indications of local maxima. Model selection followed the criteria [31]: (1) lower values of the Akaike Information Criterion (AIC), Bayesian Information Criterion (BIC), and sample-size adjusted BIC (SSA-BIC) indicated better model fit; (2) a significant *p*-value for the Lo–Mendell–Rubin Test (LMR) and the Bootstrap Likelihood Ratio Test (BLRT) suggested that the model with k profiles fits better than the k–1 model; (3) higher entropy values (closer to 1) indicated greater classification accuracy. Additionally, average posterior probabilities, the smallest class size, model parsimony, and interpretability were considered in determining the optimal solution. In subsequent analyses, the latent class variable was dummy-coded and included as an independent variable.

Then, R3STEP was used to analyze the covariates, and the BCH method was applied to the analysis of SA and NSSI [31, 32].

To isolate the associations of the rumination–hopelessness profiles after controlling for covariates, hierarchical logistic regression was conducted to further examine their effects on SA and NSSI. Variables were entered in three steps using a general linear model (GLM) framework. In Model 1, demographic variables (gender, age, and grade) were included as covariates. Model 2 added affective disorders (depression and state anxiety) to Model 1. Model 3 further included rumination–hopelessness latent profiles. Categorical variables were converted into factors with designated reference categories. For each model, regression coefficients (β), standard errors (SE), *p*-values, and odds ratios (ORs) were reported. Model fit was compared across the three nested models using the Likelihood Ratio Test (LRT). A significant difference in deviance (*p* < 0.05) indicated that the added variables provided incremental explanatory power.

## Results

### Common method biases

Harman’s one-factor test was used. The results showed that the total number of factors with eigenvalues greater than 1 was 12, and the total variance explained by the first common factor was 24.76%. Therefore, there was no serious common method bias in the measurement data of this study.

### Demographic differences and descriptive statistics

The life-time prevalence of SA was 12.41% (95% CI 10.30%–14.51%), and the life-time prevalence rate of NSSI was 7.26% (95% CI 5.61%–8.91%). Given the relatively low rate of self-harm behaviors, we included a brief precision assessment in our analytic strategy. To ensure transparency regarding precision and the expected uncertainty, all key estimates from the R3STEP analysis, BCH comparisons, and hierarchical logistic regression models were reported with 95% confidence intervals. Descriptive statistics and bivariate correlations are presented in Table 1. The results indicated that both rumination and hopelessness were significantly correlated with anxiety, depression, SA and NSSI. In addition, demographic variables, including age, grade, and gender, were also associated with SA. Grade and gender were associated with NSSI. Therefore, these demographic variables were included as covariates in the subsequent analyses of variance.

**Table 1:** Descriptive statistics and bivariate correlations

	1	2	3	4	5	6	7	8	9	10
1.Age	1									
2.Grade	0.29**	1								
3.Gender	0.03	− 0.02	1							
4.BHS	0.08*	0.11**	− 0.01	1						
5.RRS	− 0.03	0.07*	− 0.21**	0.32**	1					
6.SAI	0.09**	0.13**	− 0.15**	0.54**	0.49**	1				
7.TAI	0.07*	0.13**	− 0.14**	0.57	0.52**	0.84**	1			
8.BDI	0.02	0.10**	− 0.18**	0.47**	0.67**	0.52**	0.55**	1		
9.SA	0.09**	0.14**	− 0.14**	0.28**	0.40**	0.32**	0.32**	0.47**	1	
10.NSSI	− 0.02	0.07*	− 0.11**	0.28**	0.39**	0.27**	0.30**	0.43**	0.46**	1
M	16.58	–	–	6.83	38.74	43.73	45.24	11.09	–	–
SD	1.01	–	–	3.60	11.99	9.06	8.36	9.54	–	–

*BHS* Beck Hopeless Scale, *RRS* Ruminative Responses Scale, *SAI* State Anxiety Inventory, *TAI* Trait Anxiety Inventory, *BDI* Beck Depression Inventory, *SA* Suicidal attempts, *NSSI* Non-suicidal self-injury, *N* Number of participants

*p < 0.05, **p < 0.01, ***p < 0.001

### Rumination–hopelessness latent profile analysis

**Fig. 1:**
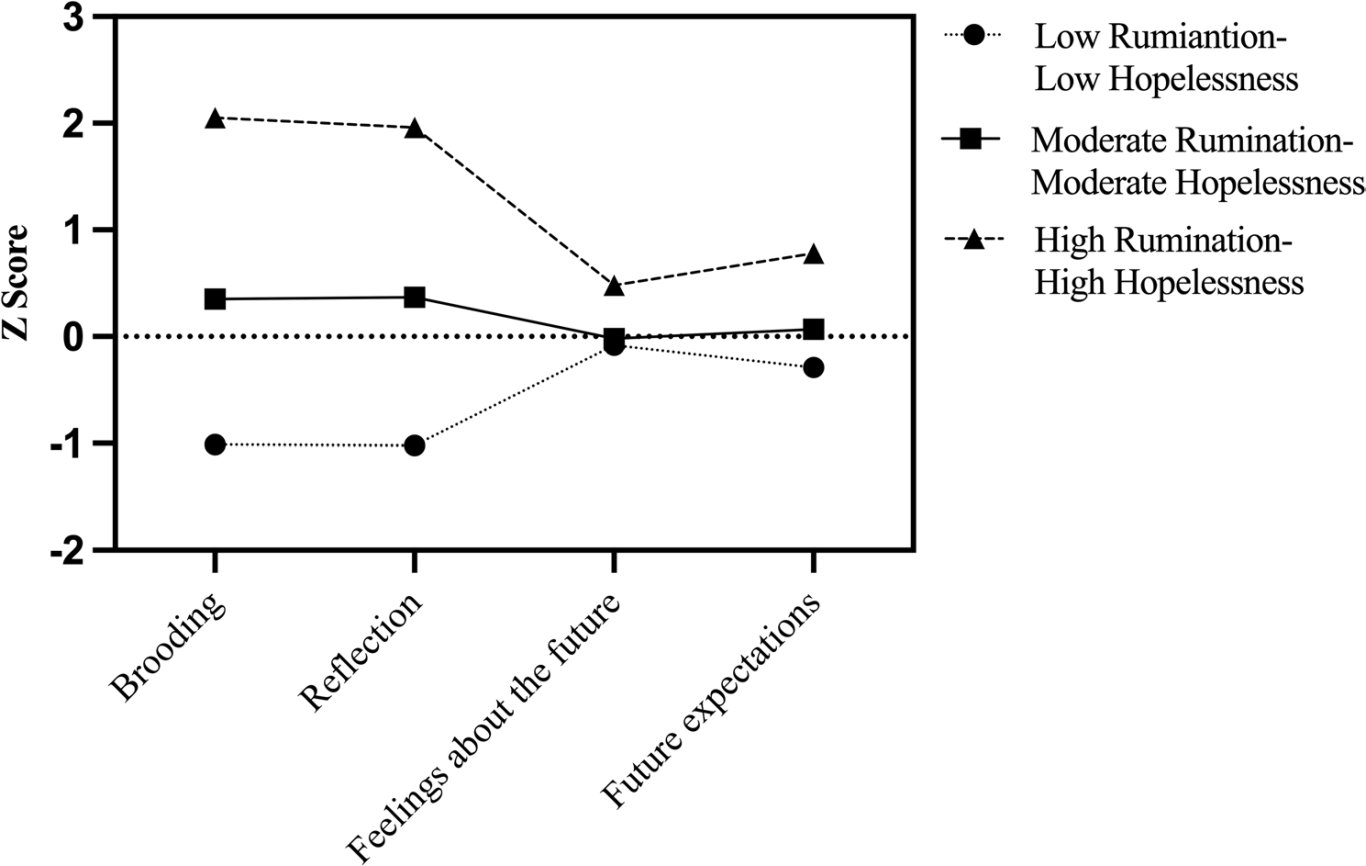
Latent profiles of rumination–hopelessness

Latent profiles were analyzed sequentially from 1 classification using the brooding dimension and the reflection dimension of the RRS scale and the feelings about the future dimension and the future expectations dimension of the BHS scale as observational variables, and the indicators of each model fit are shown in Table 2. And we can see the average posterior probabilities for latent profiles in Table 3. The results showed that the AIC, BIC, and SSA-BIC values gradually decreased with each additional classification. Starting with three classifications, the entropy values were all greater than 0.8, indicating that the classification accuracy of three, four, and five classifications were all greater than 90%. Considering that profiles need to represent a minimum of 5% of the overall sample, and taking into account model simplicity and interpretability, the 3-classification model was the best-fitting model (Table 2—model 3, Fig. 1). The average posterior probabilities for all three latent classes exceeded 0.90, indicating good classification quality (Table 3). Class 1 was characterized by low levels of rumination and average levels of hopelessness, and was labeled the “low rumination–low hopelessness” group (*n* = 343, 36.07%). Class 2 exhibited average levels of both rumination and hopelessness, and was labeled the “moderate rumination–moderate hopelessness” group (*n* = 531, 55.84%). Class 3 was marked by high levels of both rumination and hopelessness, and was labeled the “high rumination–high hopelessness” group (*n* = 77, 8.09%). Table 4 shows the differences in the scores of the three categories on the observed variables. ANOVA results revealed significant differences (*p* < 0.05) among the groups on the RRS brooding, RRS reflection, BHS feelings about the future, and BHS future expectations variables. Post hoc analyses showed that the high rumination–high hopelessness group scored higher than the other two groups on each item. The moderate rumination–moderate hopelessness group scored higher than the low rumination-low hopelessness group on all three observed variables (RRS brooding, RRS reflection, and BHS future expectations) but did not differ from the low rumination-low hopelessness group on BHS feelings about the future.

**Table 2:** Rumination–hopelessness latent profile analysis fitting indicators (*n* = 951)

Model	AIC	BIC	SSA-BIC	Entropy	LMR (*p*)	BLRT (*p*)	Proportion of potential categories	Group size
1	10,807.28	10,846.14	10,820.74	–	–	–	–	–
2	10,197.56	10,260.71	10,219.42	0.76	< 0.001	< 0.001	0.45/0.55	424/527
3	9540.31	9627.74	9570.58	0.90	< 0.001	< 0.001	0.36/0.56/0.08	343/531/77
4	9404.42	9516.14	9443.10	0.90	0.03	< 0.001	0.34/0.10/0.04/0.52	322/99/35/495
5	9296.40	9432.42	9343.49	0.89	0.02	< 0.001	0.23/0.07/0.40/0.27/0.03	223/69/370/257/32

*AIC* Akaike Information Criterion, *BIC* Bayesian Information Criterion, *SSA-BIC* sample size-adjusted BIC, *LRT* Lo-Mendell-Rubin likelihood ratio test, *BLRT* Bootstrap likelihood ratio test, *Entropy* information entropy

**Table 3:** Average posterior probabilities for latent profiles

Model	Proportion of potential categories	Average posterior probabilities
1	–	–
2	0.45/0.55	Class 1 = 0.93; Class 2 = 0.94
3	0.36/0.56/0.08	Class 1 = 0.96; Class 2 = 0.96; Class 3 = 0.94
4	0.34/0.10/0.04/0.52	Class 1 = 0.96; Class 2 = 0.89; Class 3 = 0.95; Class 4 = 0.94
5	0.23/0.07/0.40/0.27/0.03	Class 1 = 0.88; Class 2 = 0.93; Class 3 = 0.93; Class 4 = 0.96; Class 5 = 0.98

**Table 4:** Differences in means of latent profiles across observed variables

Observed variables	Low rumination–low hopelessness	Moderate rumination–moderate hopelessness	High rumination–high hopelessness	F	Post hoc	η_*p*_^2^
Brooding	− 1.01 (0.02)	0.35 (0.02)	2.05 (0.07)	1637.73***	(3) > (2) > (1)	0.78
Reflection	− 1.02 (0.02)	0.37 (0.02)	1.96 (0.08)	1524.80***	(3) > (2) > (1)	0.76
Feelings about the future	− 0.08 (0.05)	− 0.02 (0.04)	0.48 (0.12)	10.20***	(3) > (2), (1)	0.02
Future expectations	− 0.29 (0.05)	0.07 (0.04)	0.78 (0.12)	42.95***	(3) > (2) > (1)	0.08

***p < 0.001

### Covariate effects on latent profiles

R3STEP was used to examine whether covariates were associated with latent profiles. Using Class 2 as reference, gender significantly differentiated Class 1 from Class 2, indicating that males were more likely to be assigned into Class 1 (Table 5).

**Table 5:** R3STEP analysis of covariates with class 2 as reference

	Class 2 as reference					
	Class 1			Class 3		
	β	SE	OR (95%CI)	β	SE	OR (95%CI)
Age	0.001	0.07	1.00 (0.89, 1.13)	− 0.16	0.14	0.86 (0.68, 1.08)
Gender	0.94***	0.16	2.56 (1.98, 3.30)	− 0.46	0.30	0.63 (0.39, 1.03)
Grade	− 0.08	0.16	0.92 (0.70, 1.20)	0.46	0.32	1.59 (0.94, 2.69)
BDI	− 0.17***	0.02	0.84 (0.82, 0.87)	0.18***	0.02	1.19 (1.15, 1.24)
TAI	− 0.10***	0.01	0.90 (0.89, 0.92)	0.23***	0.04	1.26 (1.19, 1.34)
SAI	− 0.08***	0.01	0.92 (0.91, 0.94)	0.22***	0.03	1.25 (1.19, 1.32)

*BDI* Beck Depression Inventory, *TAI* Trait Anxiety Inventory, *SAI* State Anxiety Inventory

Class 1 = low rumination–low hopelessness group, Class 2 = moderate rumination–moderate hopelessness group, Class 3 = high rumination–high hopelessness group. *p < 0.05, **p < 0.01, ***p < 0.001. OR, Odds Ratio (OR > 1: Increased risk; OR < 1: Decreased risk; OR = 1: No effect)

For affective disorders, higher levels of depression, trait anxiety, and state anxiety decreased the likelihood of being assigned to Class 1 relative to Class 2, but increased the likelihood of being assigned to Class 3 relative to Class 2 (Table 5).

To evaluate the robustness of the findings, we repeated the analyses using a four-class model, which also showed good fit (Table 2). The R3STEP analysis from the four-class model indicated that gender and all affective symptoms (depression, trait anxiety, and state anxiety) were associated with profile membership in patterns consistent with those observed in the three-class model (Table S5).

### Results of BCH pairwise comparisons

The BCH approach was applied to examine the associations of the rumination–hopelessness profiles with SA and NSSI, accounting for classification uncertainty. Pairwise comparisons of the predicted probabilities across the three classes showed that all contrasts were significant (Table 6). The probability of both SA and NSSI increased monotonically across profiles, following the pattern Class 1 < Class 2 < Class 3.

**Table 6:** BCH analysis for probabilities of SA/NSSI

	SA/NSS I = 1 Probability (95% CI)	OR (95% CI)		
		Class 1 vs. Class 2	Class 1 vs. Class 3	Class 2 vs. Class 3
SA	Class 1 = 0.04 (0.02, 0.06)	0.37*** (0.21, 0.63)	0.03*** (0.02, 0.06)	0.09*** (0.06, 0.15)
	Class 2 = 0.11 (0.09, 0.14)			
	Class 3 = 0.57 (0.47, 0.67)			
NSSI	Class 1 = 0.003 (− 0.004, 0.01)	0.04*** (0.003, 0.48)	0.004*** (0.00,0.06)	0.12*** (0.07,0.20)
	Class 2 = 0.07 (0.05, 0.09)			
	Class 3 = 0.40 (0.30, 0.49)			

*SA* Suicidal attempts, *NSSI* Non-suicidal self-injury

Class 1 = low rumination–low hopelessness group, Class 2 = moderate rumination–moderate hopelessness group, Class 3 = high rumination–high hopelessness group. *p < 0.05, **p < 0.01, ***p < 0.001. OR, Odds Ratio (OR > 1: Increased risk; OR < 1: Decreased risk; OR = 1: No effect)

On the BCH approach was also applied to four-class sensitivity model. Pairwise comparisons of probabilities revealed a clear severity gradient across groups (Table S6). Class 1 showed the lowest probability of SA, significantly lower than Classes 2 and 3, and its NSSI probability was lower than Class 3. Class 2 demonstrated higher SA and NSSI probabilities than Class 4, but lower than Class 3. Class 3 exhibited the highest probabilities of both SA and NSSI compared with all other classes.

### Hierarchical logistic regression of latent classes on SA and NSSI

Model 2 significantly improved model fit compared to Model 1 (Δχ²_(2)_ = 180.20, *p* < 0.001), indicating that depression and anxiety were significantly associated with SA. Model 3 also showed improvement over Model 2 (Δχ²_(2)_ = 6.23, *p* = 0.04), suggesting that the rumination–hopelessness latent categories contributed additional explanatory power for SA.

**Fig. 2:**
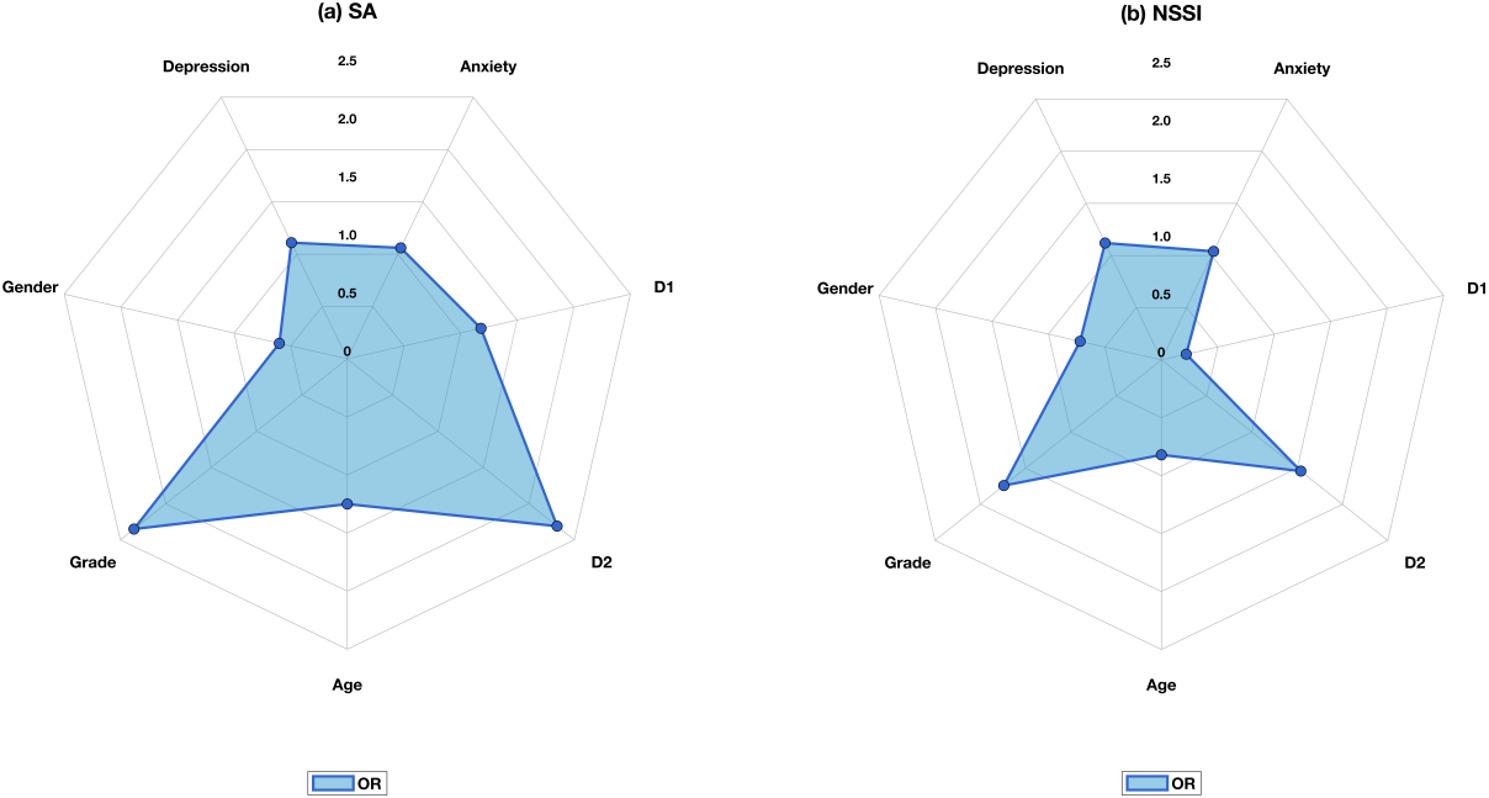
Odds Ratios from hierarchical logistic regression analyses. *SA* Suicidal attempts, *NSSI* Non-suicidal self-injury. Categorical variable coding (D1: 0 = moderate rumination–moderate hopelessness group, 1 = low rumination–low hopelessness group; D2: 0 = moderate rumination–moderate hopelessness group, 1 = high rumination–high hopelessness group). OR, Odds Ratio (OR > 1: Increased risk; OR < 1: Decreased risk; OR = 1: No effect)

Age, grade, and gender were used as covariates in predicting SA. The results of the hierarchical model for SA are shown in Table 7, with estimates reported as β coefficients, standard errors, and odds ratios with 95% confidence intervals. In Model 1, grade (β = 1.03, SE = 0.29, OR = 2.79, 95%CI 1.59–4.90, *p* < 0.001) and gender (β = − 0.91, SE = 0.22, OR = 0.40, 95%CI 0.26–0.62, *p* < 0.001) were significantly associated with SA, and the difference in age did not reach statistical significance (*p* = 0.09). Females exhibited a higher prevalence of SA than males, and a higher level of grade was associated with an increased incidence of SA. Model 2 with the addition of state anxiety and depression found that state anxiety (β = 0.07, SE = 0.02, OR = 1.07, 95%CI 1.03–1.12, *p* < 0.001) and depression (β = 0.11, SE = 0.01, OR = 1.12, 95%CI 1.09–1.15, *p* < 0.001) were significantly associated with SA. Higher levels of anxiety and depression were associated with an increased risk of SA. Dummy variables were created for the rumination–hopelessness latent profiles (D1: 0 = moderate rumination–moderate hopelessness, 1 = low rumination–low hopelessness; D2: 0 = moderate rumination–moderate hopelessness, 1 = high rumination–high hopelessness). After controlling for variables in the first two steps, the high rumination–high hopelessness group (β = 0.84, SE = 0.34, OR = 2.31, 95%CI 1.20–4.45, *p* = 0.01, *p*_FDR = 0.02) showed a significantly higher risk of SA compared to the moderate rumination–moderate hopelessness group. No significant difference was found between the low rumination–low hopelessness group and the moderate rumination–moderate hopelessness group (*p* = 0.63, *p_*FDR = 0.63). In Model 3, age (β = 0.22, SE = 0.11, OR = 1.25, 95%CI 1.02–1.54, *p* = 0.03, *p*_FDR = 0.046) was significant associated with SA, while the effect of gender was no longer statistically significant (*p* = 0.05, *p*_FDR = 0.06). The regression coefficients are presented in Fig. 2.

**Table 7:** Hierarchical regression analysis of SA and NSSI (Class 2 as reference)

Model	Variables	SA			NSSI		
		β (SE)	OR (95%CI)	*P/P_*FDR	β (SE)	OR (95%CI)	*P/P_*FDR
Model 1	Age	0.16 (0.09)	1.17 (0.98, 1.41)	0.09	− 0.18 (0.14)	0.84 (0.64, 1.10)	0.20
	Grade	1.03 (0.29)	2.79 (1.59, 4.90)	< 0.001	0.77 (0.33)	2.16 (1.13, 4.11)	0.02
	Gender	− 0.91 (0.22)	0.40 (0.26, 0.62)	< 0.001	− 0.93 (0.29)	0.40 (0.23, 0.70)	0.001
Model 2	Age	0.20 (0.10)	1.23 (1.00, 1.50)	0.05	− 0.24 (0.16)	0.79 (0.57, 1.09)	0.15
	Grade	0.85 (0.33)	2.34 (1.22, 4.50)	0.01	0.54 (0.39)	1.72 (0.80, 3.68)	0.16
	Gender	− 0.51 (0.26)	0.60 (0.36, 0.998)	0.049	− 0.52 (0.33)	0.60 (0.31, 1.15)	0.12
	Depression	0.11 (0.01)	1.12 (1.09, 1.15)	< 0.001	0.13 (0.02)	1.14 (1.10, 1.18)	< 0.001
	Anxiety	0.07 (0.02)	1.07 (1.03, 1.12)	< 0.001	0.05 (0.02)	1.05 (1.00, 1.10)	0.04
Model 3	Age	0.22 (0.11)	1.25 (1.02, 1.54)	0.03/0.046	− 0.20 (0.17)	0.82 (0.59, 1.14)	0.23/ 0.31
	Grade	0.85 (0.34)	2.35 (1.21, 4.57)	0.01/0.02	0.55 (0.39)	1.74 (0.81, 3.74)	0.16/ 0.31
	Gender	− 0.52 (0.27)	0.60 (0.35, 1.01)	0.05/0.06	− 0.32 (0.34)	0.72 (0.37, 1.41)	0.34/ 0.34
	Depression	0.11 (0.02)	1.11 (1.08, 1.14)	< 0.001/< 0.001	0.12 (0.02)	1.12 (1.08, 1.17)	< 0.001/ < 0.001
	Anxiety	0.06 (0.02)	1.06 (1.02, 1.11)	0.004/0.01	0.04 (0.03)	1.04 (0.989, 1.09)	0.13/ 0.31
	D1	0.16 (0.34)	1.18 (0.60, 2.30)	0.63/0.63	− 1.51 (0.76)	0.22 0.05, 0.98)	0.046/ 0.194
	D2	0.84 (0.34)	2.31 (1.20, 4.45)	0.01/0.02	0.43 (0.37)	1.54 (0.74, 3.19)	0.25/ 0.31

*SA* Suicidal attempts,*NSSI* Non-suicidal self-injury

Class 2 = moderate rumination–moderate hopelessness group, Categorical variable coding (D1: 0 = moderate rumination–moderate hopelessness group, 1 = low rumination–low hopelessness group; D2: 0 = moderate rumination–moderate hopelessness group, 1 = high rumination–high hopelessness group). OR, Odds Ratio (OR > 1: Increased risk; OR < 1: Decreased risk; OR = 1: No effect)

Model 2 significantly improved model fit compared to Model 1(Δχ²_(2)_ = 139.40, *p* < 0.001), indicating that depression and anxiety showed significant associations with NSSI. Model 3 also showed improvement over Model 2 (Δχ²_(2)_ = 7.42, *p* = 0.02), suggesting that the rumination–hopelessness latent classes contributed additional explanatory power for NSSI.

Age, grade, and gender were included as covariates in the NSSI model. The hierarchical model results examining associations with NSSI were presented in Table 7. In Model 1, both grade (β = 0.77, SE = 0.33, OR = 2.16, 95%CI 1.13–4.11, *p* = 0.02) and gender (β = − 0.93, SE = 0.29, OR = 0.40, 95%CI 0.23–0.70, *p* = 0.001) were significantly associated with NSSI. However, after including additional variables in Models 2 and 3, the associations of grade and gender with NSSI were no longer statistically significant. In Model 2, both depression (β = 0.13, SE = 0.02, OR = 1.14, 95%CI 1.10–1.18, *p* < 0.001) and anxiety (β = 0.05, SE = 0.02, OR = 1.05, 95%CI 1.00–1.10, *p* = 0.04) were significantly associated with NSSI. In Model 3, depression showed significant association with NSSI, while the effect of anxiety was no longer statistically significant (*p* = 0.13, *p*_FDR = 0.31). Neither the low rumination–low hopelessness group (D1: *p* = 0.046, *p*_FDR = 0.19) nor the high rumination–high hopelessness group (D2: *p* = 0.25, *p*_FDR = 0.31) showed significant differences with the moderate rumination–moderate hopelessness group on NSSI. The regression coefficients are presented in Fig. 2.

We also conducted sensitivity analyses of TAI. In the hierarchical logistic regression analyses, we replaced SAI with TAI to evaluate the stability of the associations when using an alternative anxiety construct. The results were highly consistent with the main analyses, indicating that the inclusion of trait anxiety did not materially alter the interpretation of group differences (Table S3).

Because the four-class also demonstrated good fit and competitive performance, we re-estimated four-class regression model. Sensitivity analysis identified that Class 3 had a significantly higher risk of SA compared with Class 4. In contrast, the differences between Class 4 and the other profiles on NSSI were not statistically significant. This pattern may reflect the reduced between-class contrasts that occur when the number of classes increases, as shown in Table S7. The overall direction of effects remained consistent across models.

## Discussion

Rumination and hopelessness are closely associated with affective disorders and self-harm behaviors. Our study used latent profile analysis (LPA) to examine the generalized association of rumination–hopelessness profiles across different types of self-harm behaviors in a non-clinical adolescent sample. Our results reveal distinct latent profiles characterized by combined features of rumination and hopelessness exhibited differential effects related to SA and NSSI, emphasizing the importance of utilizing LPA to capture complex cognitive co-occurrence. More specifically, the high rumination–high hopelessness profile was uniquely associated with a highly likelihood of SA. This association remained significant after statistically controlling for general affective symptoms. In contrast, NSSI was significantly linked to the severity of depressive symptoms, indicating less specificity in its association with the cognitive latent profiles.

### Latent profiles of rumination–hopelessness among adolescents and their characteristics

Our study identified three latent profiles through LPA: the low rumination–low hopelessness group, the moderate rumination–moderate hopelessness group, and the high rumination–high hopelessness group. These groups exhibited significant and distinct step-wise differences across the severity of rumination and hopelessness, confirming that the combination of these cognitive traits varies meaningfully in our adolescent sample. The clustering of high levels of rumination and hopelessness in the same profile is consistent with prior research showing their frequent co-occurrence in adolescents. This interrelationship may be explained by the reciprocal relationship between rumination and hopelessness. Individuals tended to engage in persistent and repetitive thinking about hopelessness-related cognitive content. This sustained focus on hopelessness may trigger further rumination, creating a feedback loop that exacerbates both constructs [33]. Additionally, rumination and hopelessness may share similar neural mechanisms. Rumination has been linked to increased functional connectivity within emotional networks, particularly positive connectivity between the amygdala and prefrontal regions. This connectivity may influence emotional regulation through comparable neural circuits [34]. In another study, depressed patients with a history of suicide were found to have significant correlations between activation in the orbital frontal cortex and the dorsolateral prefrontal cortex. This activation was associated with feelings of hopelessness and may be similar to rumination, involving the over processing of negative emotional information in the prefrontal cortex [35].

### Interpretation of covariate and distal outcome analyses

Our analysis using the R3STEP approach revealed important findings about the factors influencing membership in the identified latent profiles. We observed a significant gradient in profile assignment based on affective symptoms: individuals with lower levels of anxiety and depression were significantly more likely to belong to the low rumination–low hopelessness profile, while those with higher affective distress were more likely to be assigned to the high rumination–high hopelessness profile (Class 3). This pattern suggests that affective distress acts as a trigger for the formation of the high-risk cognitive profile, fostering the simultaneous enhancement of rumination and hopelessness [36–38].

Furthermore, we found that gender significantly influenced profile assignment, with males being more likely to be classified into the lower-risk group (Class 1) relative to the moderate-risk group (Class 2). This is consistent with existing literature indicating that adolescent females generally exhibit higher levels of rumination [39], supporting that established demographic differences in key cognitive traits contribute to the divergence into distinct self-harm risk profiles.

Using the BCH method to account for classification uncertainty, we established a clear link between the latent cognitive profiles and the distal outcomes of SA and NSSI. The probability of engaging in both SA and NSSI increased progressively and significantly across the profiles, following a precise severity gradient (Class 1 < Class 2 < Class 3). This finding supports previous research demonstrating that high levels of rumination and hopelessness are individually associated with heightened self-harm risk [15, 16, 18, 19]. Crucially, our study extends this by demonstrating that the co-occurrence of high rumination and high hopelessness forms a combined, high-risk cognitive profile that is systematically linked to the heightened likelihood of self-harm behaviors. This systematic gradient underscores the clinical utility of identifying this specific, extreme cognitive signature when assessing self-harm risk in adolescents.

### Specific effects of rumination–hopelessness latent profiles on SA and NSSI

Our hierarchical logistic regression analyses revealed that the latent profiles of rumination and hopelessness had different effects on SA and NSSI. The most compelling finding is the robust association between the high rumination–high hopelessness profile and SA risk, independent of affective distress. Previous meta-analytic evidence indicated that overall rumination and brooding rumination were moderately positively associated with SA, whereas the reflection showed no significant association with SA [40]. In our study, differences in both brooding and reflection dimensions were observed across latent profiles, suggesting that overall rumination may be associated with SA. Both retrospective and prospective analyses have consistently found that levels of hopelessness were significantly higher in individuals with a history of suicide behaviors compared to those without such a history [41]. Our study extends these findings by characterizing the combination of high rumination and high hopelessness as a dual cognitive constriction in SA. Individuals with co-occurrence of dual cognitive rigidity and despair may develop a tunnel vision, perceiving death as the only viable solution to their crisis [42], ultimately leading to the escalation of more lethal forms of self-harm such as SA [43]. The specific cognitive vulnerability in SA extends beyond general affective symptoms, implying that it may serve as a mechanism that translates affective distress into suicidal behaviors [44].

In contrast to SA, the likelihood of NSSI in our sample was strongly associated with the severity of depressive symptoms, but not uniquely related to the complex cognitive profiles. This finding supports the dominant theoretical understanding of NSSI. NSSI was considered a “hidden” form of self-harm behaviors that occurred below the surface of the iceberg—common in the community but often unreported and without help-seeking behaviors—and typically served as a means for adolescents to quickly escape pain or regulate emotions [43]. Because the primary goal of NSSI is immediate relief, its occurrence may be more closely tied to the intensity of general negative affect captured by the continuous depressive symptom scores rather than the temporally rigid, future-oriented hopelessness that characterizes high-risk SA profiles. This highlights a fundamental functional and motivational divergence between the two forms of self-harm [45].

Moreover, the significant effect of grade observed in our analyses suggested that the risk of suicide behaviors was higher among students in upper grades, potentially reflecting both ongoing cognitive development and increasing academic pressure associated with higher educational levels. This is consistent with previous evidence indicating a strong correlation between academic stress and self-harm behaviors, mediated in part by internalizing symptoms such as anxiety and depression [46].

### Limitations

This study has several limitations that warrant cautious interpretation of the findings. Firstly, the cross-sectional nature of the data restricts our ability to establish temporal or causal relationships. Future studies using clinician-administered assessments and longitudinal designs are needed to further validate the predictive utility of these profiles across different developmental and clinical contexts. Secondly, although we demonstrated the suitability of the Chinese-adapted measures, the reliance on self-report and single-item assessment for SA and NSSI may introduce misclassification bias. Future studies are encouraged to consider the use of multi-item scales to improve measurement fidelity and diagnostic accuracy. Thirdly, while formal testing suggested common method bias was not severe, we recognize that method variance remains a potential limitation, possibly leading to inflated associations between self-report constructs. Future studies should employ multi-modal assessment strategies to ensure the independence and accurate estimation. Moreover, the use of a non-clinical adolescent sample limits the generalizability of the findings to clinical populations, where self-harm rates and comorbidity levels are substantially higher.

## Conclusion

Our study employed a person-centered LPA approach to explore combined features of rumination and hopelessness, two cognitive factors closely associated with adolescent self-continuity. The findings suggest that a specific, severe cognitive profile (high rumination–high hopelessness) is robustly and uniquely associated with SA, whereas NSSI is primarily related to the intensity of depressive symptoms. Our study resolves a key theoretical gap in self-harm mechanism by mapping cognitive phenotypes to differential self-harm pathways. Moreover, our work indicates actionable taxonomy that enables pre-symptomatic screening via rumination/hopelessness assessment and reorients intervention toward cognitive-specific targets alongside traditional affective management.

## Supplementary Information

Below is the link to the electronic supplementary material.

Supplementary Material 1.

## Data Availability

Due to ethical restrictions, the data are not publicly available. Anonymized data may be shared upon reasonable request and institutional approval.
