# Supplementary material for: Latent patterns of rumination and hopelessness on self-harm behaviors in adolescence
Source: Child Adolesc Psychiatry Ment Health. 2026 Jan 29;20:77. doi: 10.1186/s13034-026-01029-0 (PMC13231778; doi:10.1186/s13034-026-01029-0)
Supplement: Supplementary file 1 — Supplementary Material 1. [file 13034_2026_1029_MOESM1_ESM.docx]

**Table**

Table S1 Multicollinearity diagnostics for logistic regression (SAI).

|  | SA |  |  | NSSI |  |
| --- | --- | --- | --- | --- | --- |
| Variables | VIF | df |  | VIF | df |
| Age | 1.10 | 1 |  | 1.14 | 1 |
| Grade | 1.08 | 1 |  | 1.14 | 1 |
| Gender | 1.07 | 1 |  | 1.04 | 1 |
| BDI | 1.47 | 1 |  | 1.48 | 1 |
| SAI | 1.29 | 1 |  | 1.45 | 1 |
| 3-Class | 1.43 | 2 |  | 1.38 | 2 |

*Note.* BDI (Beck Depression Inventory), SAI (State Anxiety Inventory). VIF, Variance inflation factor.

Table S2 Multicollinearity diagnostics for logistic regression (TAI).

|  | SA |  |  | NSSI |  |
| --- | --- | --- | --- | --- | --- |
| Variables | VIF | df |  | VIF | df |
| Age | 1.10 | 1 |  | 1.15 | 1 |
| Grade | 1.08 | 1 |  | 1.14 | 1 |
| Gender | 1.07 | 1 |  | 1.05 | 1 |
| BDI | 1.55 | 1 |  | 1.52 | 1 |
| TAI | 1.40 | 1 |  | 1.45 | 1 |
| 3-Class | 1.43 | 2 |  | 1.34 | 2 |

*Note.* BDI (Beck Depression Inventory), TAI (Trait Anxiety Inventory). VIF, Variance inflation factor.

Table S3 Hierarchical regression analysis of SA and NSSI (Class2 as reference, TAI).

|  |  | SA | | |  | NSSI | | |
| --- | --- | --- | --- | --- | --- | --- | --- | --- |
| Model | Variables | β (SE) | OR  (95%CI) | *P/*  *P_*FDR |  | β (SE) | OR  (95%CI) | *P/*  *P_*FDR |
| Model 1 | Age | 0.16 (0.09) | 1.17  (0.98, 1.41) | 0.09 |  | -0.18 (0.14) | 0.84  (0.64, 1.10) | 0.21 |
|  | Grade | 1.03 (0.29) | 2.79  (1.59, 4.90) | <0.001 |  | 0.77 (0.33) | 2.16  (1.13, 4.11) | 0.02 |
|  | Gender | -0.91 (0.22) | 0.40  (0.26, 0.62) | <0.001 |  | -0.93 (0.29) | 0.40  (0.23, 0.70) | 0.001 |
|  |  |  |  |  |  |  |  |  |
| Model 2 | Age | 0.22 (0.11) | 1.24  (1.01, 1.52) | 0.04 |  | -0.22 (0.17) | 0.81  (0.58, 1.11) | 0.19 |
|  | Grade | 0.78 (0.33) | 2.19  (1.15, 4.17) | 0.02 |  | 0.49 (0.39) | 1.63  (0.76, 3.50) | 0.21 |
|  | Gender | -0.52 (0.26) | 0.59  (0.36, 0.98) | 0.04 |  | -0.49 (0.33) | 0.62  (0.32, 1.19) | 0.15 |
|  | Depression | 0.12 (0.01) | 1.12  (1.09, 1.16) | <0.001 |  | 0.13 (0.02) | 1.13  (1.09, 1.18) | <0.001 |
|  | Anxiety | 0.06 (0.02) | 1.06  (1.01, 1.10) | 0.01 |  | 0.06 (0.03) | 1.07  (1.01, 1.12) | 0.02 |
|  |  |  |  |  |  |  |  |  |
| Model 3 | Age | 0.24 (0.11) | 1.27  (1.03, 1.56) | 0.03/  0.04 |  | -0.19 (0.17) | 0.83  (0.59, 1.15) | 0.25/  0.29 |
|  | Grade | 0.80 (0.34) | 2.22  (1.15, 4.29) | 0.02/  0.04 |  | 0.50 (0.39) | 1.66  (0.77, 3.58) | 0.20/  0.29 |
|  | Gender | -0.53 (0.27) | 0.59  (0.35, 0.99) | 0.045/  0.05 |  | -0.29 (0.34) | 0.75  (0.38, 1.46) | 0.40/  0.40 |
|  | Depression | 0.11 (0.02) | 1.11  (1.08, 1.15) | <0.001/  <0.001 |  | 0.11 (0.02) | 1.12  (1.07, 1.16) | <0.001/  <0.001 |
|  | Anxiety | 0.05 (0.02) | 1.05  (1.01, 1.10) | 0.03/  0.04 |  | 0.05 (0.03) | 1.05  (1.00, 1.11) | 0.05/  0.14 |
|  | D1 | 0.18 (0.34) | 1.20  (0.61, 2.34) | 0.59/  0.59 |  | -1.48 (0.76) | 0.23  (0.05, 1.01) | 0.05/  0.14 |
|  | D2 | 0.92 (0.33) | 2.50  (1.31, 4.79) | 0.01/  0.02 |  | 0.44 (0.37) | 1.55  (0.75, 3.19) | 0.23/  0.29 |

*Note.* SA (Suicidal attempts), NSSI (Non-suicidal self-injury). Class2 = Moderate Rumination-Moderate Hopelessness group, Categorical variable coding (D1: 0 = Moderate Rumination-Moderate Hopelessness group, 1 = Low Rumination-Low Hopelessness group; D2: 0 = Moderate Rumination-Moderate Hopelessness group, 1 = High Rumination-High Hopelessness group). OR, Odds Ratio (OR > 1: Increased risk; OR < 1: Decreased risk; OR = 1: No effect).

Table S4 Differences in means of four classes across observed variables.

| Observed variables | Class1 | Class2 | Class3 | Class4 | F | Post hoc | η_p_^2^ |
| --- | --- | --- | --- | --- | --- | --- | --- |
| Brooding | -1.07 (0.02) | 1.37 (0.04) | 2.56(0.10) | 0.24 (0.02) | 1819.82*** | (1)<(4)<(2)<(3) | 0.85 |
| Reflection | -1.04 (0.02) | 1.22 (0.06) | 2.43 (0.11) | 0.26 (0.02) | 1077.19*** | (1)<(4)<(2)<(3) | 0.77 |
| Feelings about the Future | -0.06 (0.06) | 0.09 (0.10) | 0.84 (0.21) | -0.04 (0.04) | 9.32*** | (1), (4), (2)<(3) | 0.03 |
| Future Expectations | -0.25 (0.06) | 0.45 (0.09) | 1.09 (0.16) | -0.01 (0.04) | 29.81*** | (1)<(4)<(2)<(3) | 0.09 |

*Note.* ****p* < 0.001. Class1 = Low Rumination-Low Hopelessness, Class2 = Moderate high Rumination-Moderate Hopelessness, Class3 = High Rumination-High Hopelessness, Class4 = Moderate Rumination-Moderate Hopelessness.

Table S5 R3STEP analysis of covariates with Class 4 as reference (four-class model).

|  | Class4 as reference | | | | | | | | | | |
| --- | --- | --- | --- | --- | --- | --- | --- | --- | --- | --- | --- |
|  | Class1 | | |  | Class2 | | |  | Class3 | | |
|  | β | SE | OR (95%CI) |  | β | SE | OR (95%CI) |  | β | SE | OR (95%CI) |
| Age | –0.02 | 0.07 | 0.98 (0.87, 1.11) |  | –0.25 | 0.14 | 0.78 (0.62, 0.99) |  | –0.16 | 0.19 | 0.85 (0.62,1.15) |
| Gender | 0.98*** | 0.16 | 2.66 (2.04, 3.46) |  | -0.33 | 0.28 | 0.72 (0.45, 1.14) |  | 0.03 | 0.38 | 1.03 (0.55, 1.92) |
| Grade | -0.06 | 0.17 | 0.94 (0.72, 1.24) |  | 0.93** | 0.36 | 2.54 (1.42, 4.56) |  | -0.03 | 0.39 | 0.97 (0.51, 1.85) |
| BDI | -0.16*** | 0.02 | 0.85 (0.83, 0.88) |  | 0.13*** | 0.02 | 1.14 (1.11, 1.17) |  | 0.31*** | 0.04 | 1.36 (1.27, 1.46) |
| TAI | -0.08*** | 0.01 | 0.92 (0.91, 0.94) |  | 0.13** | 0.04 | 1.14 (1.06, 1.22) |  | 0.32*** | 0.06 | 1.38 (1.26, 1.51) |
| SAI | -0.06*** | 0.01 | 0.94 (0.92, 0.95) |  | 0.11*** | 0.03 | 1.11 (1.05, 1.17) |  | 0.31*** | 0.05 | 1.36 (1.26, 1.47) |

*Note.* BDI (Beck Depression Inventory), TAI (Trait Anxiety Inventory), SAI (State Anxiety Inventory). Class1 = Low Rumination-Low Hopelessness, Class2 = Moderate high Rumination-Moderate Hopelessness, Class3 = High Rumination-High Hopelessness, Class4 = Moderate Rumination-Moderate Hopelessness. **p* < 0.05, ***p* < 0.01, ****p* < 0.001. OR, Odds Ratio (OR > 1: Increased risk; OR < 1: Decreased risk; OR = 1: No effect).

Table S6 BCH analysis for probabilities of SA/NSSI (four-class model).

|  | SA/NSSI=1 Probability (95% CI) | OR(95% CI) | | | |
| --- | --- | --- | --- | --- | --- |
|  |  | Class1 | Class2 | Class3 | Class4 |
| SA | Class1 = 0.04 (0.02, 0.06) | 0 | 0.09*  (0.05, 0.17) | 0.01***  (0.004, 0.03) | 0.47  (0.26, 0.84) |
|  | Class2 = 0.32 (0.24, 0.41) |  | 0 | 0.12***  (0.05,0.29) | 5.10***  (3.00, 8.70) |
|  | Class3 = 0.80 (0.67, 0.92) |  |  | 0 | 41.67***  (18.52, 90.91) |
|  | Class4 = 0.09 (0.06, 0.11) |  |  |  | 0 |
| NSSI | Class1 = 0.004 (-0.003, 0.01) | 0 | 0.02  (0.003, 0.12) | 0.003***  (0.00, 0.02) | 0.07  (0.01, 0.47) |
|  | Class2 = 0.18 (0.11, 0.26) |  | 0 | 0.16***  (0.07, 0.34) | 3.86***  (2.03, 7.35) |
|  | Class3 = 0.59 (0.45, 0.74) |  |  | 0 | 25.00***  (12.35, 50.00) |
|  | Class4 = 0.06 (0.04,0.07) |  |  |  | 0 |

*Note.* SA (Suicidal attempts), NSSI (Non-suicidal self-injury). Class1 = Low Rumination-Low Hopelessness, Class2 = Moderate high Rumination-Moderate Hopelessness, Class3 = High Rumination-High Hopelessness, Class4 = Moderate Rumination-Moderate Hopelessness. **p* < 0.05, ***p* < 0.01, ****p* < 0.001. OR, Odds Ratio (OR > 1: Increased risk; OR < 1: Decreased risk; OR = 1: No effect).

Table S7 Hierarchical regression analysis of SA and NSSI (Class4 as reference, four-class model).

|  |  | SA | | |  | NSSI | | |
| --- | --- | --- | --- | --- | --- | --- | --- | --- |
| Model | Variables | β (SE) | OR  (95%CI) | *P/*  *P_*FDR |  | β (SE) | OR  (95%CI) | *P/*  *P_*FDR |
| Model 1 | Age | 0.16 (0.09) | 1.17  (0.98, 1.41) | 0.09 |  | -0.18 (0.14) | 0.84  (0.64, 1.10) | 0.21 |
|  | Grade | 1.03 (0.29) | 2.79  (1.59, 4.90) | <0.001 |  | 0.77 (0.33) | 2.16  (1.13, 4.11) | 0.02 |
|  | Gender | -0.91 (0.22) | 0.40  (0.26, 0.62) | <0.001 |  | -0.93 (0.29) | 0.40  (0.23, 0.70) | 0.001 |
|  |  |  |  |  |  |  |  |  |
| Model 2 | Age | 0.20 (0.10) | 1.23  (0.999, 1.50) | 0.05 |  | -0.24 (0.16) | 0.79  (0.57, 1.09) | 0.15 |
|  | Grade | 0.85 (0.33) | 2.34  (1.22, 4.50) | 0.01 |  | 0.54 (0.39) | 1.72  (0.80, 3.68) | 0.16 |
|  | Gender | -0.51 (0.26) | 0.60  (0.36, 0.998) | 0.049 |  | -0.52 (0.33) | 0.60  (0.31, 1.15) | 0.12 |
|  | Depression | 0.11 (0.01) | 1.12  (1.09, 1.15) | <0.001 |  | 0.13 (0.02) | 1.14  (1.10, 1.18) | <0.001 |
|  | Anxiety | 0.07 (0.02) | 1.07  (1.03, 1.12) | <0.001 |  | 0.05 (0.02) | 1.05  (1.00, 1.10) | 0.04 |
|  |  |  |  |  |  |  |  |  |
| Model 3 | Age | 0.24 (0.11) | 1.27  (1.03, 1.56) | 0.03/  0.04 |  | -0.21 (0.17) | 0.81  (0.58, 1.14) | 0.23/  0.32 |
|  | Grade | 0.94 (0.36) | 2.55  (1.26, 5.13) | 0.01/  0.02 |  | 0.62 (0.40) | 1.85  (0.84, 4.07) | 0.13/  0.29 |
|  | Gender | -0.58 (0.27) | 0.56  (0.33, 0.96) | 0.04/  0.046 |  | -0.40 (0.35) | 0.67  (0.34, 1.33) | 0.25/  0.32 |
|  | Depression | 0.09 (0.02) | 1.10  (1.07, 1.13) | <0.001/  <0.001 |  | 0.11 (0.02) | 1.12  (1.08, 1.17) | <0.001/  <0.001 |
|  | Anxiety | 0.06 (0.02) | 1.06  (1.01, 1.10) | 0.01/  0.02 |  | 0.03 (0.03) | 1.04  (0.986, 1.09) | 0.17/  0.30 |
|  | D1 | 0.09 (0.36) | 1.09  (0.54, 2.19) | 0.81/  0.81 |  | -1.42 (0.76) | 0.24  (0.05, 1.07) | 0.06/  0.27 |
|  | D2 | 0.55 (0.31) | 1.73  (0.94, 3.20) | 0.08/  0.09 |  | 0.09 (0.38) | 1.09  (0.52, 2.27) | 0.82/  0.82 |
|  | D3 | 1.87 (0.56) | 6.50  (2.18, 19.30) | <0.001/  0.002 |  | 0.90 (0.53) | 2.46  (0.87, 7.00) | 0.09/  0.27 |

*Note.* SA (Suicidal attempts), NSSI (Non-suicidal self-injury). Class4 = Moderate Rumination-Moderate Hopelessness group, Categorical variable coding (D1: 0 = Class2, Class3, Class4, 1 = Class1; D2: 0 = Class1, Class3, Class4, 1 = Class2; D3: 0 = Class1, Class2, Class4, 1 = Class3). OR, Odds Ratio (OR > 1: Increased risk; OR < 1: Decreased risk; OR = 1: No effect).

Table S8 Multicollinearity diagnostics for logistic regression (four-class model).

|  | SA |  |  | NSSI |  |
| --- | --- | --- | --- | --- | --- |
| Variables | VIF | df |  | VIF | df |
| Age | 1.11 | 1 |  | 1.16 | 1 |
| Grade | 1.11 | 1 |  | 1.17 | 1 |
| Gender | 1.08 | 1 |  | 1.06 | 1 |
| BDI | 1.50 | 1 |  | 1.61 | 1 |
| SAI | 1.28 | 1 |  | 1.46 | 1 |
| 4-Class | 1.57 | 3 |  | 1.65 | 3 |

*Note.* BDI (Beck Depression Inventory), SAI (State Anxiety Inventory). VIF, Variance inflation factor.

**Figure**


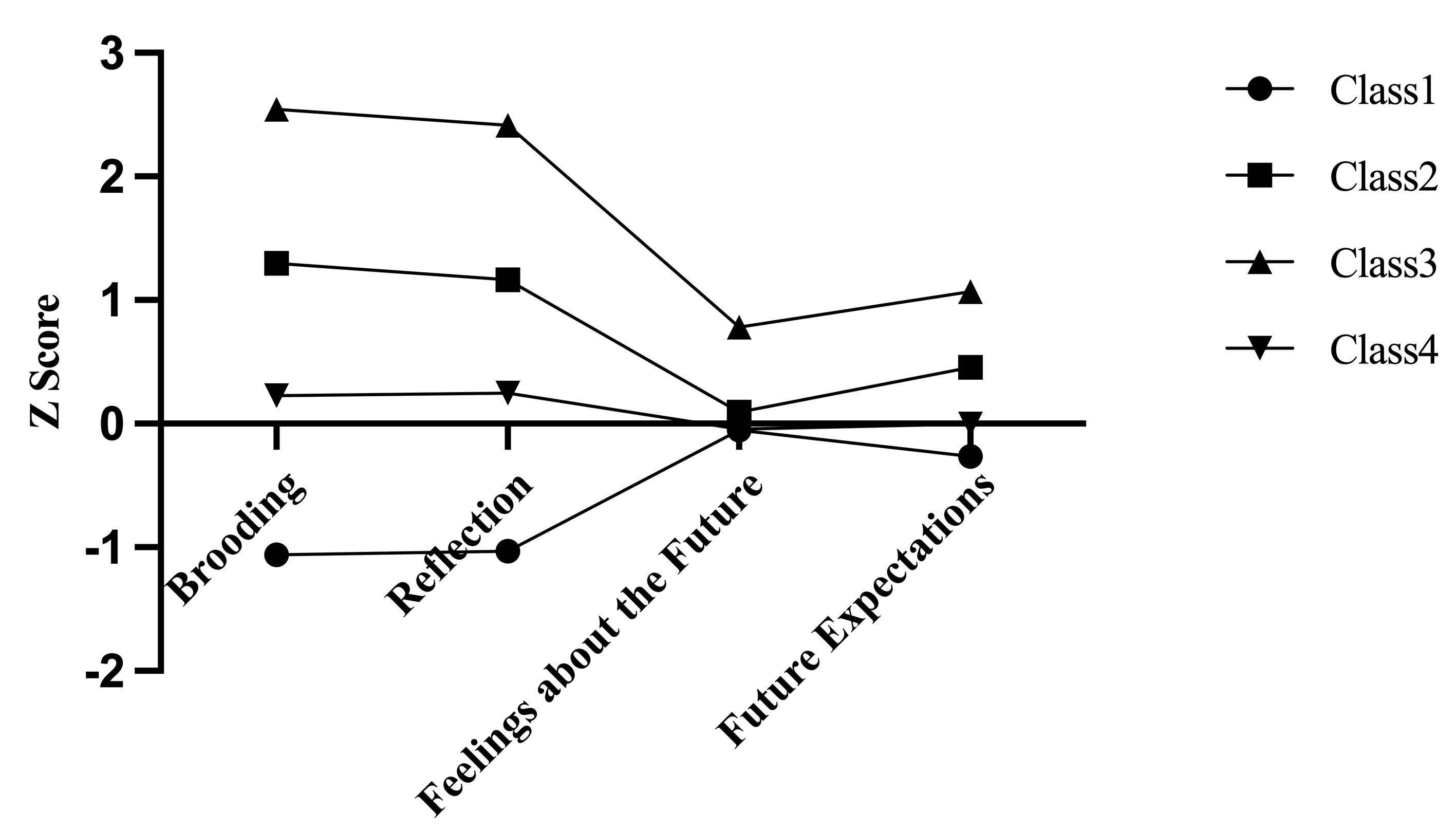


Figure S1 Latent profiles of Rumination-Hopelessness (four-class model).

*Note.* Class1 = Low Rumination-Low Hopelessness, Class2 = Moderate high Rumination-Moderate Hopelessness, Class3 = High Rumination-High Hopelessness, Class4 = Moderate Rumination-Moderate Hopelessness.


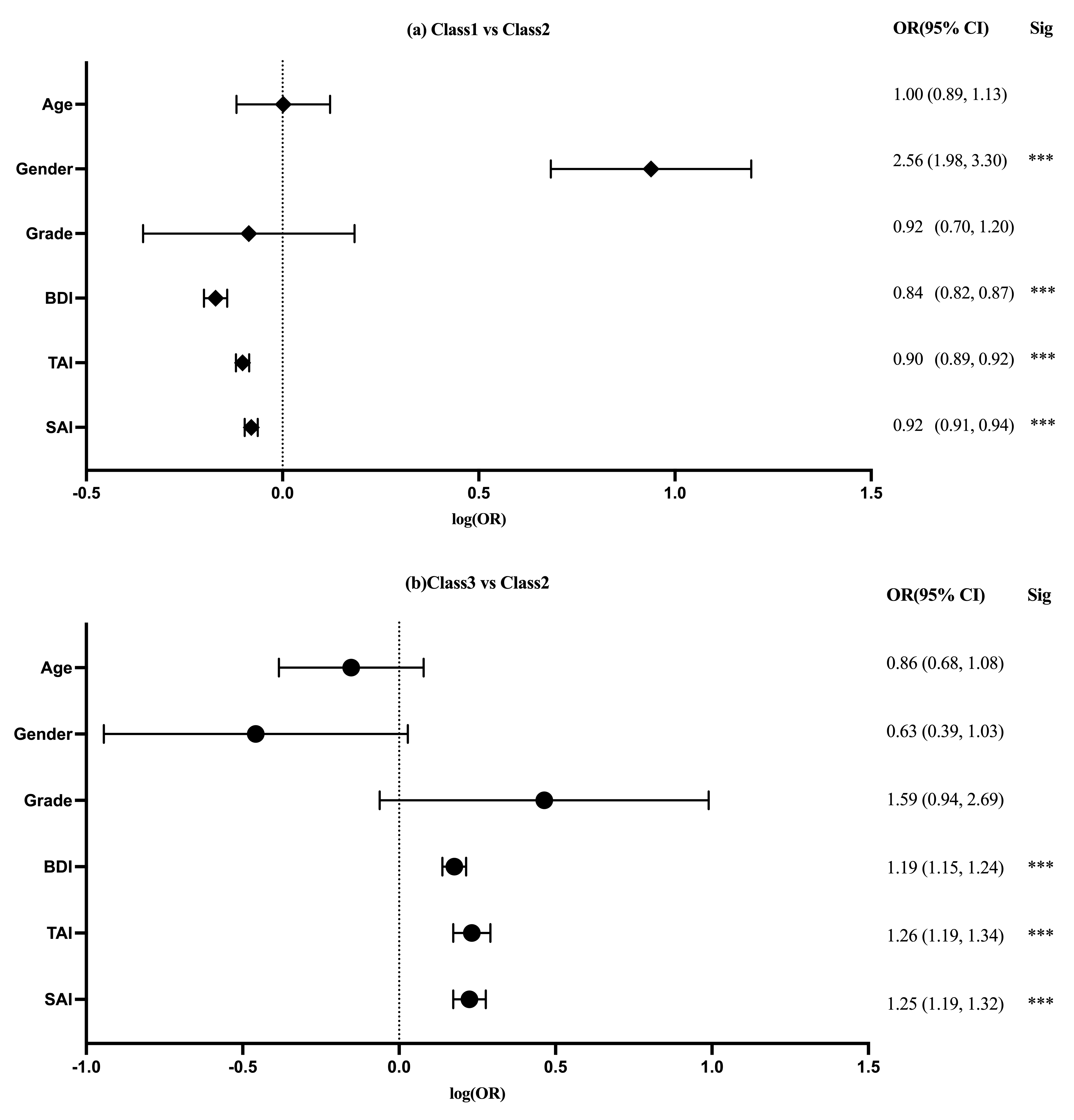


Figure S2 Forest plot for three-class R3STEP analysis

*Note.* BDI (Beck Depression Inventory), TAI (Trait Anxiety Inventory), SAI (State Anxiety Inventory). Class1 = Low Rumination-Low Hopelessness group, Class2 = Moderate Rumination-Moderate Hopelessness group, Class3 = High Rumination-High Hopelessness group. *p < 0.05, **p < 0.01, ***p < 0.001. OR, Odds Ratio (log(OR) > 0: Increased risk; log(OR) < 0: Decreased risk; log(OR) = 0: No effect).


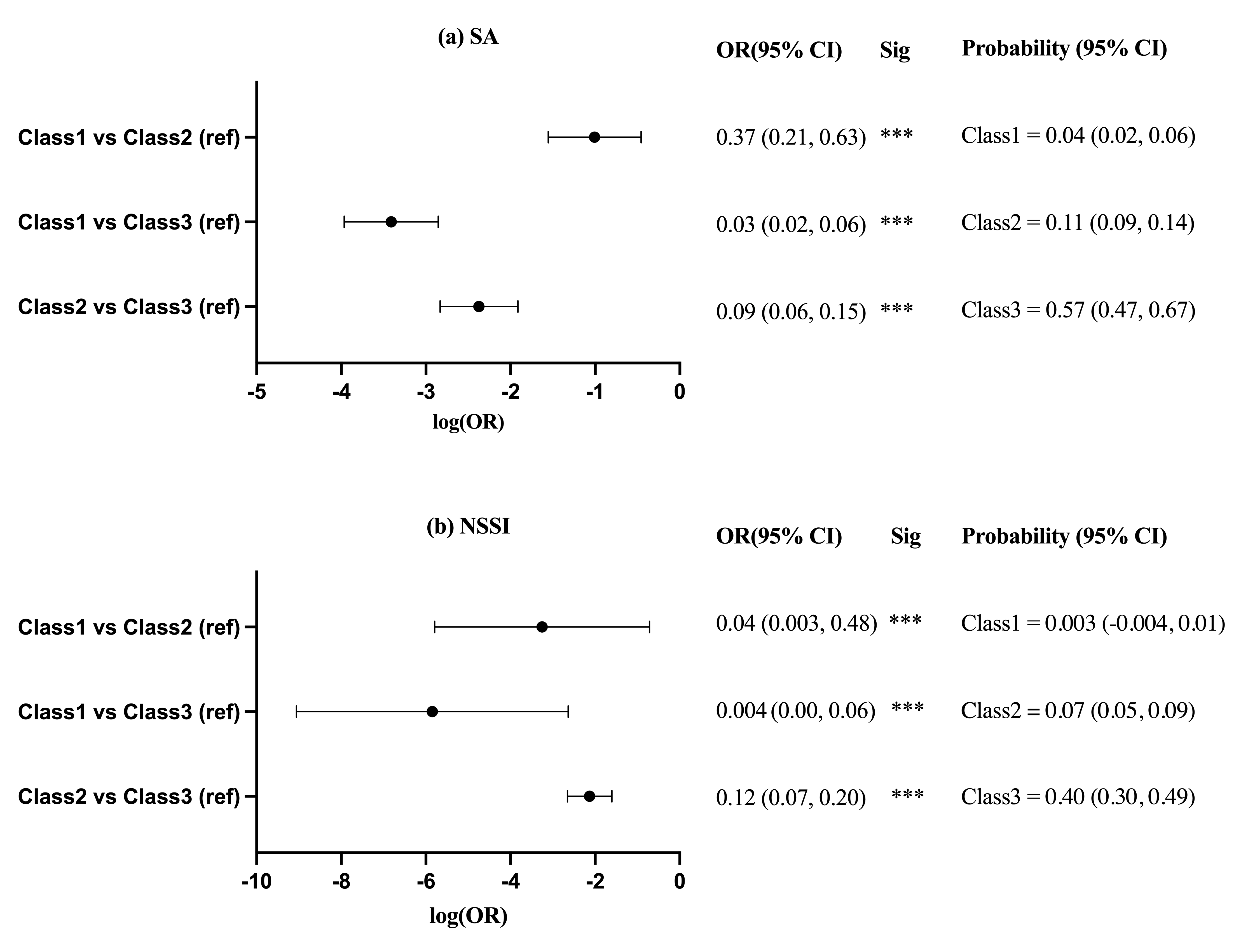


Figure S3 Forest plot for three-class BCH analysis

*Note.* Class1 = Low Rumination-Low Hopelessness group, Class2 = Moderate Rumination-Moderate Hopelessness group, Class3 = High Rumination-High Hopelessness group. *p < 0.05, **p < 0.01, ***p < 0.001. OR, Odds Ratio (log(OR) > 0: Increased risk; log(OR) < 0: Decreased risk; log(OR) = 0: No effect).
